# Arylbenzazepines Are Potent Modulators for the Delayed Rectifier K^+^ Channel: A Potential Mechanism for Their Neuroprotective Effects

**DOI:** 10.1371/journal.pone.0005811

**Published:** 2009-06-05

**Authors:** Xue-Qin Chen, Jing Zhang, John L. Neumeyer, Guo-Zhang Jin, Guo-Yuan Hu, Ao Zhang, Xuechu Zhen

**Affiliations:** 1 State Key laboratory of Drug Research, Department of Pharmacology II, Shanghai Institute of Materia Medica, Chinese Academy of Sciences, Shanghai, China; 2 Synthetic Organic & Medicinal Chemistry Laboratory (SOMCL), Shanghai Institute of Materia Medica, Chinese Academy of Sciences, Shanghai, China; 3 Alcohol and Drug Abuse Research Center, McLean Hospital, Harvard Medical School, Belmont, Massachusetts, United States of America; National Cancer Institute, United States of America

## Abstract

(±) SKF83959, like many other arylbenzazepines, elicits powerful neuroprotection *in vitro* and *in vivo*. The neuroprotective action of the compound was found to partially depend on its D_1_-like dopamine receptor agonistic activity. The precise mechanism for the (±) SKF83959-mediated neuroprotection remains elusive. We report here that (±) SKF83959 is a potent blocker for delayed rectifier K^+^ channel. (±) SKF83959 inhibited the delayed rectifier K^+^ current (*I*_K_) dose-dependently in rat hippocampal neurons. The *IC*_50_ value for inhibition of *I*_K_ was 41.9±2.3 µM (Hill coefficient = 1.81±0.13, n = 6), whereas that for inhibition of *I*_A_ was 307.9±38.5 µM (Hill coefficient = 1.37±0.08, n = 6). Thus, (±) SKF83959 is 7.3-fold more potent in suppressing *I*_K_ than *I*_A_. Moreover, the inhibition of *I*_K_ by (±) SKF83959 was voltage-dependent and not related to dopamine receptors. The rapidly onset of inhibition and recovery suggests that the inhibition resulted from a direct interaction of (±) SKF83959 with the K^+^ channel. The intracellular application of (±) SKF83959 had no effects of on *I*_K_, indicating that the compound most likely acts at the outer mouth of the pore of K^+^ channel. We also tested the enantiomers of (±) SKF83959, R-(+) SKF83959 (MCL-201), and S-(−) SKF83959 (MCL-202), as well as SKF38393; all these compounds inhibited *I*_K_. However, (±) SKF83959, at either 0.1 or 1 mM, exhibited the strongest inhibition on the currents among all tested drug. The present findings not only revealed a new potent blocker of *I*_K_ , but also provided a novel mechanism for the neuroprotective action of arylbenzazepines such as (±) SKF83959.

## Introduction

**Figure 1:**
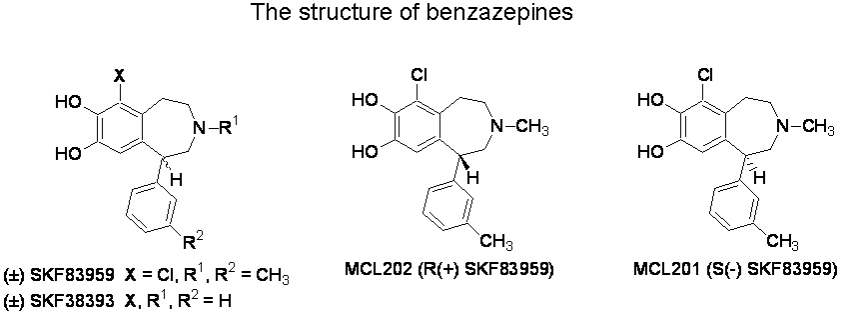
The structures of benzazepines.

Atypical D_1_ receptor agonist (±) SKF83959, (±) 3-methyl-6-chloro-7,8-hydroxy-1-(3-methylphenyl)-2,3,4,5-tetrahydro-1H-3-benzazepine (Fig. 1), possesses a unique pharmacological property. (±) SKF83959 does not stimulate cAMP formation in brain tissues but activates the phosphatidylinositol (PI)-linked pathway via a D_1_-like dopamine receptors (D_1_DAR) [1]–[5], although it produces D_1_ agonist-mediated behavioral responses in animals [6]. This compound was shown to have anti-parkinsonian effect in an experimental primate model as well as in the unilateral lesioned rodent model for Parkinson's disease [7]–[11]. Earlier studies with (±) SKF83959 and its isomers, showed that such D_1_-like agonists increase eye blinking in monkeys [12] and rats [13] and that the magnitude of this effect may be related to agonistic efficacy. Although there is a recent report by employing a different approach depicting that (±) SKF83959 is able to stimulate native brain tissues coupling to both Gs and Gq protein[14], the antiparkinsonian mechanism of the agent, however, is known not to relate to the cAMP pathway and may associate with the (±) SKF83959-mediated PI-linked D_1_DAR activation [3], [6], [15]–[17]. In agreement with a previous report in a model of primate Parkinson's disease, in which (±) SKF83959 not only reduced the dyskinesias but also reduced the occurrence of motor fluctuation and wearing-off dyskinesia [15], we have recently demonstrated that chronic treatment of (±) SKF83959 resulted in a significantly lower dyskinesia while eliciting its potent anti-parkinsonian action in a model of 6-hydroxydopamine (6-OHDA) unilaterally -lesioned rats. Moreover, a chronic administration of (±) SKF83959 also significantly reduced levo-DOPA-induced dyskinesias in PD rats [18]. The underlying mechanism for the anti-dyskinesia action of (±) SKF83959 is believed to associate with the drug's powerful neuroprotective action [19]. Since it was suggested that occurrence of motor fluctuation and wearing-off dyskinesia are associated with anti-PD drug treatment-induced further loss of dopaminergic neurons [20], [21]. We recently demonstrated that in both cultured neurons and in HEK293 cells, (±) SKF83959 inhibited GSK3β activity via a D_1_-like receptor-dependent mechanism. However, blockage of D_1_-like receptor activation which blunted SKF83959-mediated inhibition on GSK3β,was found to attenuated partially the neuroprotective effect of (±) SKF83959 [19], indicating that D_1_-like receptor-independent mechanism may be also involved in the neuroprotection of the drug.

Delayed rectifier K^+^ channel plays an important role in neuroprotection [22]–[24]. It has been proposed that decreased intracellular K^+^ due to activation of the K^+^ channel contributes to the neuroapoptosis induced by various insults such as Aβ-amyloid [22], [25] and serum deprivation [23]. Tetraethylammonium (TEA), a classical blocker of delayed rectifier K^+^ channel, was found to exert potent neuroprotective effects *in vivo* [26], [27] and *in vitro* [23]. Considering the importance of delayed rectifier K^+^ channel in neuroprotection, we decided to explore the potential effect of (±) SKF83959 on delayed rectifier K^+^ channel as part of our efforts in understanding the mechanism for the D_1_-receptor independent neuroprotection of the drug. We here demonstrate that (±) SKF83959 and related benzazepines are potent inhibitors of delayed rectifier K^+^ channel. The potency of (±) SKF83959 is almost 25-fold higher than that of TEA. Our findings not only revealed a new category of potent blockers of delayed rectifier K^+^ channel but also provided a novel mechanism for the neuroprotective action of arylylbenzazepines analogs.

## Materials and Methods

### Materials

(±) SKF38393, (±)-SCH23390 hydrochloride, prazosin and mesulergine were purchased from Sigma (St Louis, MO, USA)/RBI (Natick, MA, USA). R-(+) SKF38393 (MCL-201) and S-(−) SKF38393 (MCL-202) (Fig. 1) were synthesized in the Alcohol and Drug Abuse Research Center at McLean Hospital (Belmont, MA, USA). (±) SKF83959 was synthesized in the Synthetic Organic & Medicinal Chemistry Laboratory, Shanghai Institute of Materia Medica, Chinese Academy of Sciences. Other chemicals were purchased from Sigma-Aldrich China Inc.

### Preparation of dissociated hippocampal neurons

All procedures were in compliance with the Guidelines for the Care and Use of Laboratory Animals (National Research Council, People's Republic of China, 1996). Dissociated hippocampal neurons were prepared from newborn (5–9 day) Sprague-Dawley rats as described previously [26]. Briefly, hippocampal slices (500 µm) were cut in oxygenated ice-cold dissociation solution consisted of the following (in mM): 82 Na_2_SO_4_, 30 K_2_SO_4_, 5 MgCl_2_, 10 HEPES, and 10 glucose, pH = 7.3 adjusted with NaOH. The slices were incubated in the dissociation solution containing protease XXIII (3 g/L) at 32°C for 8 min, and then placed in dissociation solution containing trypsin inhibitor type II-S (1 g/L) and bovine serum albumin (1 g/L) at 24–25°C under an oxygen atmosphere. Before recording, the CA1 region of several slices was dissected, and triturated using a series of fire-polished Pasteur pipettes with decreasing tip diameters. Dissociated neurons were placed in a recording dish and superfused with an external solution consisted of the following (in mM): 135 NaCl, 5 KCl, 1 CaCl_2_, 2 MgCl_2_, 10 HEPES, 10 glucose, 0.001 tetrodotoxin , pH = 7.4 adjusted with NaOH..

### Whole-cell voltage clamp recoding

Voltage-activated K^+^ currents were recorded in large pyramidal-shaped neurons using an Axopatch 200A amplifier (Axon Instruments, USA) at 24–25°C [26]. Voltage protocols were controlled by pClamp 9.0 software via a DigiData-1322A interface (Axon Instruments, USA). Electrodes (a tip resistance of 3–5 MΩ) were pulled from borosilicate grass pipettes (Sutter Instruments, USA) and filled with a pipette solution consisted of the following (in mM): 140 KCl, 2 MgCl_2_, 1 CaCl_2_, 10 HEPES, 10 EGTA (pH 7.4 with KOH). The neurons were held at −50 mV. Unless otherwise mentioned, the total K^+^ current (*I*_total_) was elicited with 400-ms depolarizing steps to +40 mV following a 600-ms hyperpolarizing prepulse to −110 mV, delivered every 10 s. The delayed rectifier K^+^ current (*I*_K_) was elicited by using a similar protocol, but a 50-ms interval at −50 mV was inserted after the prepulse to inactivate the fast transient K^+^ current (*I*_A_). *I*_A_ is the subtraction of *I*_K_ from *I*_total_. Signals were filtered at 2–10 KHz and sampled at frequencies of 10–40 KHz. Series resistance was compensated by 75%–85%. Linear leak and residual capacitance currents were subtracted online using a P/4 protocol.

### Drug application

(±) SKF83959 and other drugs were dissolved in dimethylsulfoxide (DMSO) to prepare a stock solution of 10 mM and stored at −20°C. Before use, the stock solutions were diluted to desired concentrations. For extracellular application, the drug-containing external solution was delivered to the neuron using RSC-100 rapid solution changer with a 9-tube head (BioLogic Co., France). DMSO (less than 0.1% in the final dilution) had no observed effect on the voltage-activated K^+^ currents (DMSO: 0.598±0.11 nA; control: 0.660±0.095 nA, n = 4; p>0.05). For intracellular dialysis, (±) SKF83959 contained in the pipette solution was diffused into the recorded neuron immediately after patch membrane ruptured [29].

### Data analysis

The peak amplitude of *I*_A_ was measured, whereas the steady-state amplitude of *I*_K_ was measured at 300 ms after the initiation of each voltage step. The decay time constants (τ) of the currents were obtained by fitting the decay time course with a mono-exponential function. The concentrations of (±) SKF83959 to yield 50% block of the K^+^ currents (*IC*_50_) were obtained by fitting normalized concentration–inhibition relationships to the equation: *I*/*I*_0_ = 1/{1+([C]/IC_50_)^n^}, where *I*_0_ and *I* are the current amplitudes measured in control and in the presence of (±) SKF83959, [C] is the concentration of (±) SKF83959 in the external solution and n is the Hill coefficient. The ratio of inhibition was calculated by using the equation: Inhibition =  (1−*I*/*I*_0_)*100%, where *I*_0_ and *I* are the current amplitude in control and in the presence of (±) SKF83959, respectively. For analyzing the voltage-dependence of steady-state activation or inactivation of *I*_K_, normalized conductance or current was plotted against the membrane potential, and fitted to the Boltzmann equation: Y = 1/{1+exp[(*V*−*V*_1/2_)/*k*]}, where Y is the normalized conductance or current, *V* is the test potential; *V*_1/2_ is the voltage at half-maximal activation or inactivation of *I*_K_; *k* is the slope factor. The time course of recovery of *I*_K_ from inactivation was fitted with a mono-exponential function: *I*/*I*_max_ = A*{1–exp[−Δt/τ]}, where *I*_max_ is the maximal current amplitude; *I* is the current after a recovery period of Δt; τ is the time constant; A is the amplitude coefficient. Data are presented as mean±S.E.M. Statistical significance was assessed using a Student's t-test except for the voltage dependence of inhibition of *I*_K_ by (±) SKF83959 where one-way ANOVA test was used, and *P*<0.05 was considered significant. All analyses were performed using the software “GraphPad Prism 4” and “Excel 2000”.

## Results

### Inhibition of voltage-activated K^+^ currents by (±) SKF83959 in hippoacampal neurons

**Figure 2:**
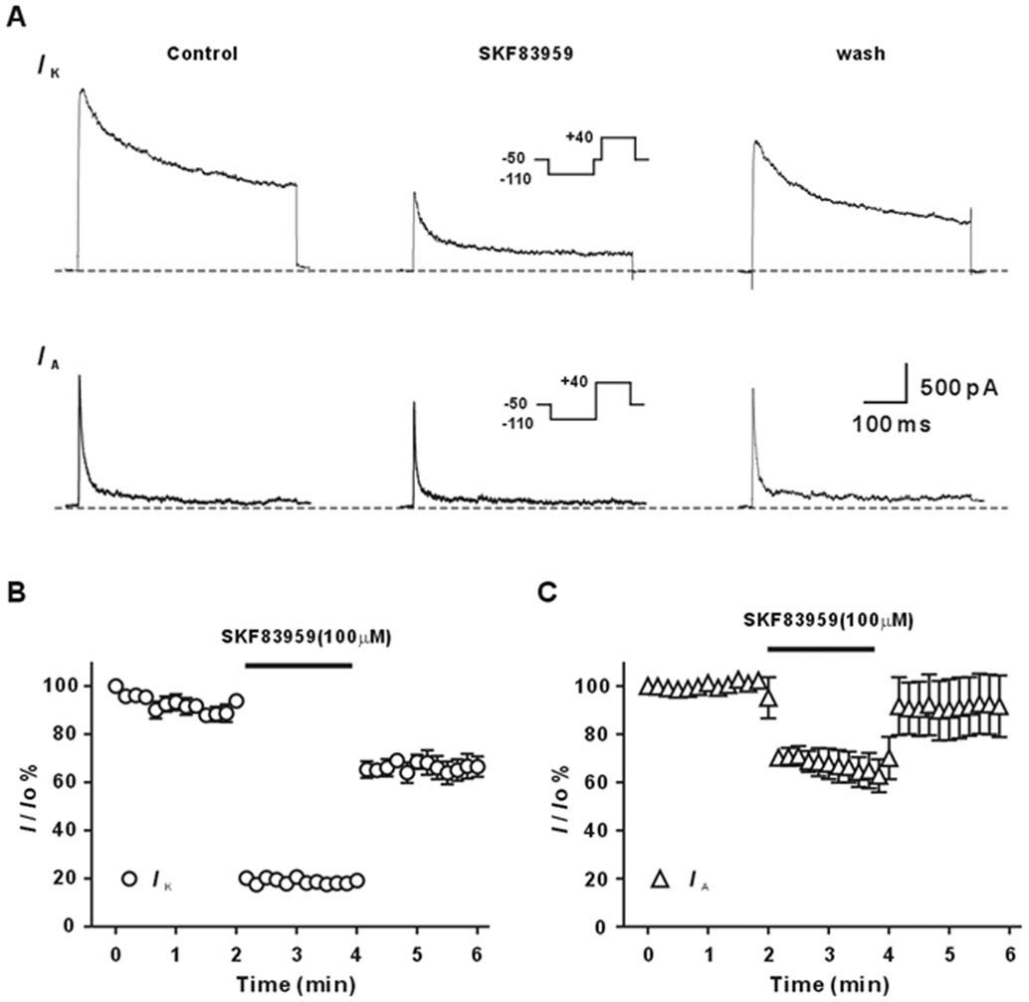
Inhibition of voltage-activated K^+^ currents by (±) SKF83959 in rat hippocampal neurons. (A) Upper and lower are the respective representative traces of the delayed rectifier K^+^ current (*I*_K_) and fast transient K^+^ current (*I*_A_) recorded prior to and during superfusion with (±) SKF83959 (100 µM) and after 10 s of washout. The neuron was held at −50 mV. Upper inset shows the pulse protocol to elicit *I*_K_, whereas lower inset shows the protocol to elicit the total K^+^ current. *I*_A_ is the subtraction of *I*_K_ from the total K^+^ current. (B) and (C) Time courses of the inhibition of *I*_K_ and *I*_A_ by (±) SKF83959 (100 µM, n = 5 for each). The bar denotes the surpufusion with SKF83959. A number of symbols in (B) and (C) have error bars smaller than their size.

Superfusion with (±) SKF83959 (10–1000 µM) inhibited both *I*_K_ and *I*_A_ in concentration-dependent manner. However, the compound preferentially inhibited *I*_K_ to *I*_A_. As shown in Fig. 2A, (±) SKF83959 (100 µM) markedly suppressed *I*_K_, whereas a moderate reduction in the amplitude of *I*_A_ was observed. The inhibition of (±) SKF83959 on both K^+^ currents had a rapid onset, as it reached steady-state levels within 10 s. Moreover, the K^+^ currents were partially recovered upon washing out the compound (Fig. 2B, 2C). The partial recovery may be because of the run-down of the currents with recording, or due to the incompletely drug washing- out.

**Figure 3:**
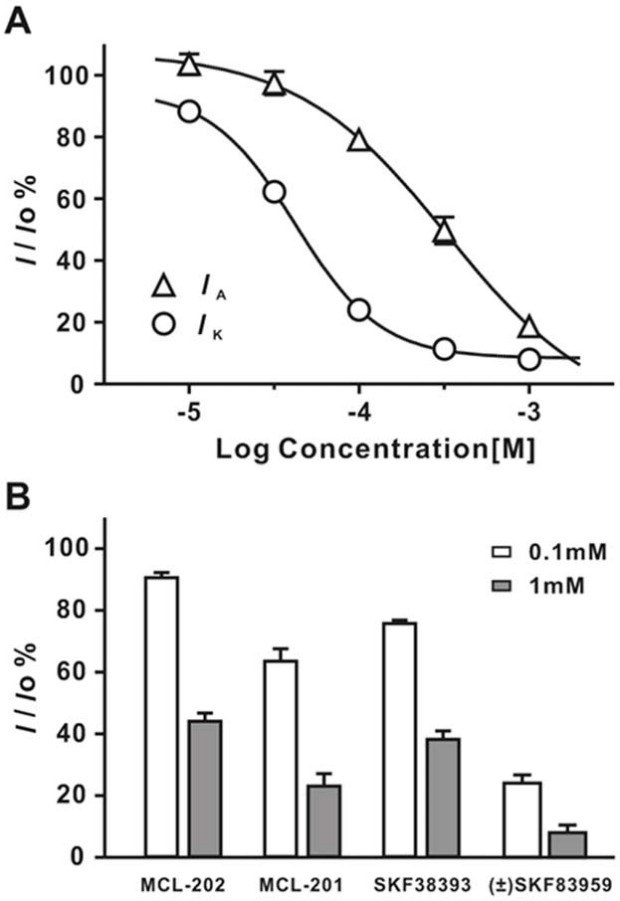
Concentration-dependent inhibition of (±) SKF83959 on the voltage-activated K^+^ currents. (A) Concentration-inhibition curves of (±) SKF83959 on the delayed rectifier K^+^ current (*I*_K_) and fast transient K^+^ current (*I*_A_)(n = 6 for *I*_K_ and *I*_A_). A number of symbols have error bars smaller than their size. (B) Comparison of the substituted phenylbenzazepine, MCL-201, MCL-202, SKF38393 and (±) SKF83959 in inhibition of *I*_K_.

Analyzing the concentration-inhibition relationships of (±) SKF83959 on K^+^ currents revealed that the *IC*_50_ value for inhibition of *I*_K_ was 41.9±2.3 µM (Hill coefficient = 1.81±0.13, n = 6), while that for inhibition of *I*_A_ was 307.9±38.5 µM (Hill coefficient = 1.37±0.08, n = 6) (Fig. 3A). Because (±) SKF83959 is 7.3-fold more potent in suppressing *I*_K_ than *I*_A_, we thus focused on characterizing the inhibition on *I*_K_.

We next examined whether the other substituted phenylbenzazepines such as R-(+) SKF83959 (MCL-201), S-(−) SKF83959 (MCL-202) and SKF38393 affect *I*_K_. The results are shown in Fig. 3B. At the concentration of 100 µM, the inhibition of *I*_K_ by MCL-201, MCL-202 and SKF38393 was 36.7±4.1% (n = 8), 9.7±1.7% (n = 8) and 24.5±1.1% (n = 7), respectively, whereas (±) SKF83959 (100 µM) induced much greater inhibition on *I*_K_ (76.0±2.7%, n = 6). At the concentration of 1 mM, the inhibition of *I*_K_ by MCL-201, MCL-202 and SKF38393 was 77.1±4.0% (n = 3), 56.1±2.7% (n = 4) and 62.0±2.7% (n = 5), respectively, whereas (±) SKF83959 (1 mM) almost completely suppress the K^+^ current (92.0±2.5%, n = 3). Thus, (±) SKF83959 produced the most potent inhibition of *I*_K_ among the four substituted benzazepines at tested concentrations.

### Mechanisms underlying the inhibition of the delayed rectifier K^+^ current by (±) SKF83959

**Figure 4:**
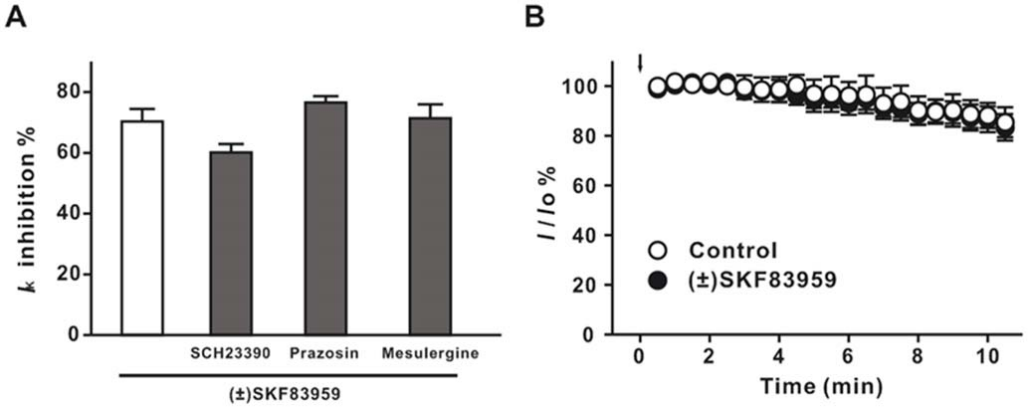
Block of the delayed rectifier K^+^ channel by (±) SKF83959 in rat hippocampal neurons. (A) Inhibition of the delayed rectifier K^+^ current (*I*_K_) by (±) SKF83959 (100 µM) in the presence of D_1_ receptor antagonist SCH23390 (20 µM, n = 6), or α1-adrenoceptor antagonist prazosin (10 µM, n = 6), or 5-HT receptor antagonist mesulergine (10 µM, n = 6). (B) Lacking effect of intracellular dialysis of (±) SKF83959 on *I*_K_ (n = 7). The recording pipettes were filled with pipette solution containing (±) SKF83959 (300 µM). The downward arrow indicates the time when the patch membrane was ruptured.

(±) SKF83959 is an atypical agonist of D_1_-like receptor. We first examined whether the inhibition of *I*_K_ by (±) SKF83959 was mediated through activation of D_1_-like receptor. Pretreatment with the D_1_ receptor antagonist SCH23390 did not significantly alter (±) SKF83959-induced inhibition on *I*_K_ (Fig. 4A). The inhibition of *I*_K_ by (±) SKF83959 (100 µM) in the presence of SCH23390 (20 µM) was 60.1±2.7% (n = 6), while that by (±±) SKF83959 alone was 70.3±4.1% (n = 7, *P* = 0.074, t = 1.97, df = 11).

In addition to D_1_-like receptor, (±) SKF83959 also exhibits moderate affinities to D_2_ receptors, α_1_-adrenoceptors and 5-HT_2A_ receptors [5]. However, neither the α1-adrenoceptors antagonist prazosin, nor the 5-HT receptor antagonist mesulergine affected (±) SKF83959-induced inhibition of *I*_K_ (Fig. 4A). The inhibition of *I*_K_ by (±) SKF83959 (100 µM) in the presence of prazosin (10 µM) or mesulergine (10 µM) was 76.6±2.1% (n = 6) and 71.4±4.6% (n = 6), respectively, which was almost identical with that of (±) SKF83959 alone treatment (70.3±4.1%, n = 7, *P* = 0.23, t = 1.29, df = 11 vs. prazosin and *P* = 0.86, t = 0.18, df = 11 vs. mesulergine). Taken together, (±) SKF83959 is a potent blocker of the delayed rectifier K^+^ channel and the inhibitory action is independent of either dopamine or serotonin receptors.

To determine the acting site of the agent on the K^+^ channel, we investigated the effect of intracellular dialysis of (±) SKF83959 on *I*_K_. The concentration for intracellular dialysis was 300 µM, which inhibited *I*_K_ by nearly 90% and is close to the value of IC50 for *I*_A_, when applied externally (Fig. 3A). Throughout the 10-min recording period, the relative amplitudes of *I*_K_ in the neurons dialyzed with (±) SKF83959 were almost identical with that of in the control group (Fig. 4B), so was the *I*_K_ (data not shown). At the end of 10-min recording, the relative amplitude of *I*_K_ in control and in the neurons dialyzed with (±) SKF83959 was 85.4±6.0% (n = 5) and 83.4±5.3% (n = 7), respectively (*P* = 0.81, t = 0.25, df = 10). The results suggest that (±) SKF83959 acts at an extracellular site of the delayed rectifier K^+^ channel.

### Voltage dependence of inhibition of the delayed rectifier K^+^ current by (±) SKF83959

**Figure 5:**
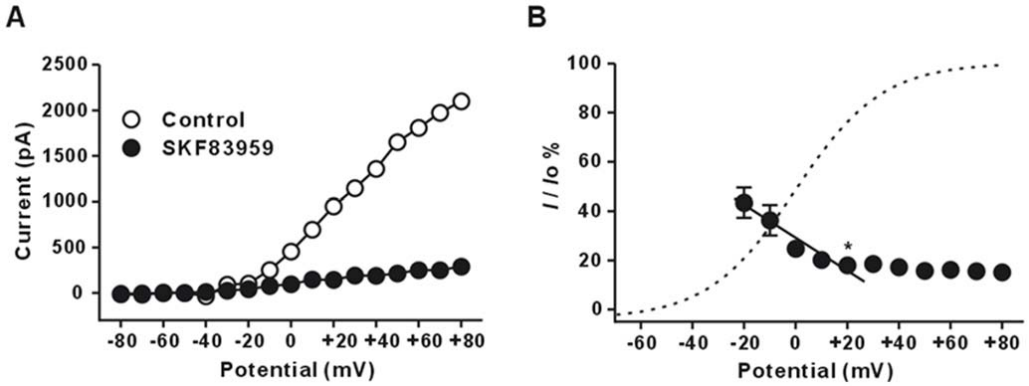
Effects of (±) SKF83959 on the current-voltage relationships of the delayed rectifier K^+^ current in rat hippocampal neurons. (A) Current-voltage (*I*/*V*) curves of the delayed rectifier K^+^ current (*I*_K_) obtained in a representative neuron in control and during superfusion of (±) SKF83959 (100 µM). (B) Plot of the relative amplitudes of *I*_K_ during superfusion of (±) SKF83959 (100 µM) as a function of the test potential. Each symbol represents the mean±S.E.M. (n = 7). The solid line is the linear fit, while the dashed line is the activation curve obtained in control condition. *P<0.01 (one-way ANOVA test) *versus* the value at −20 mV.

The current-voltage (*I*/*V*) relationship of *I*_K_ from a representative neuron in control and in the presence of (±) SKF83959 (100 µM) was plotted in Fig. 5A. (±) SKF83959 did not significantly change the threshold for activation of *I*_K_, but caused a remarkable downward shift of the *I*/*V* curve, and reduced its amplitude over the entire period of activation. The inhibition of *I*_K_ by (±) SKF83959 seems to be greater at more depolarizing potentials. Thus, we plotted the relative amplitudes of *I*_K_ as a function of the test potential. As shown in Fig. 5B, the inhibition of *I*_K_ by (±) SKF83959 steeply increased between −20 mV and +20 mV. In the presence of (±) SKF83959, the relative currents in steps to −20 mV and to +20 mV, respectively, were 43.5±6.1% and 18.1±1.8% (n = 7, *P* = 0.0019, t = 3.96, df = 12). Between +20 mV and +80 mV the inhibition remained relatively constant. The results demonstrated that the inhibition of *I*_K_ by (±) SKF83959 was voltage-dependent.

### Effects of (±) SKF83959 on kinetic behaviors of the delayed rectifier K^+^ current

**Figure 6:**
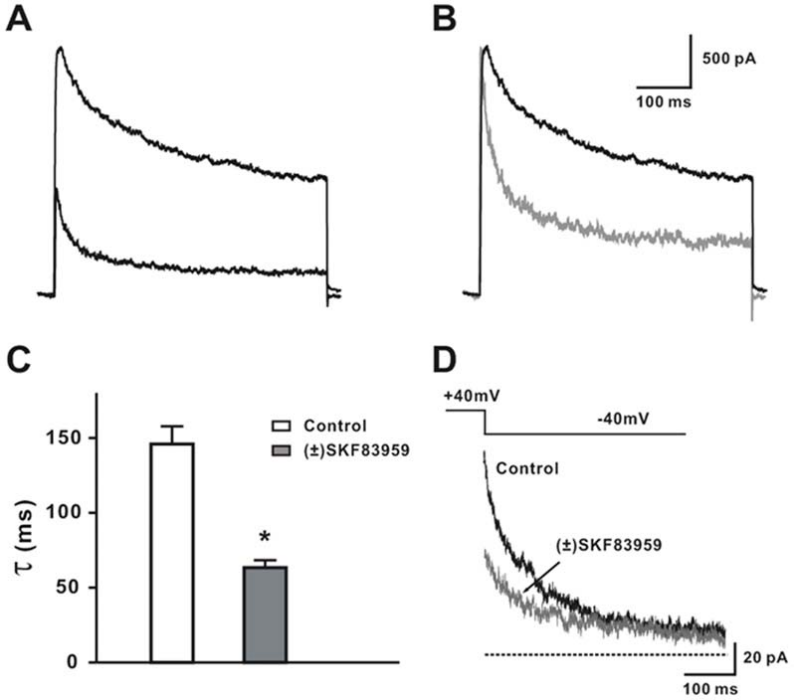
Effects of (±) SKF83959 on the decay of the delayed rectifier K^+^ current in rat hippocampal neurons. (A) Superimposed traces of the delayed rectifier K^+^ current (*I*_K_) in a representative neuron prior to and during superfusion with (±) SKF83959 (100 µM). (B) The trace with (±) SKF83959 in (A) is scaled up. (C) Decay time constants of *I*_K_ prior to and during superfusion with (±) SKF83959 (100 µM). n = 7, *P<0.01 *versus* the control. (D) Tail current traces evoked at −40 mV after a 500-ms depolarizing steps to +40 mV prior to and during superfusion with (±) SKF83959 (100 µM). Similar results were obtained in 3 neurons.

**Figure 7:**
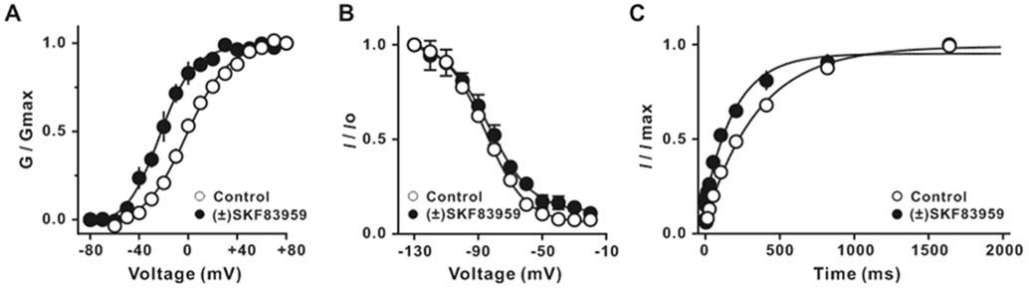
Effects of (±) SKF83959 on the activation and steady-state inactivation of the delayed rectifier K^+^ current in rat hippocampal neurons. (A) Activation curves of the delayed rectifier K^+^ current (*I*_K_) prior to and during superfusion with 100 µM (±) SKF83959 (n = 8). (B) Steady-state inactivation curves of *I*_K_ prior to and during superfusion with 100 µM (±) SKF83959 (n = 8). (C) Time courses of recovery of *I*_K_ from inactivation prior to and during superfusion with 100 µM (±) SKF83959 (n = 6). For studying the activation, neurons were held at −50 mV, currents were elicited with a series of 400-ms step from −80 mV to +80 mV in 10 mV increments following a 600-ms hyperpolarizing prepulse to −110 mV and a 50-ms interval at −50 mV, delivered every 10 s. For studying the steady-state inactivation, neurons were held at 0 mV, currents were elicited with a series of 600-ms prepulses at different hyperpolarizing potentials followed by a 50-ms interval at −50 mV and a 400-ms step to +40 mV, then back to 0 mV, delivered every 10 s. For studying the time course of recovery from inactivation, neurons were held at 0 mV, currents were elicited on return from hyperpolarizing prepulses of varying durations at −110 mV to +40 mV, delivered every 10 s.

In addition to suppressing the amplitude of *I*_K_, (±) SKF83959 (100 µM) markedly accelerated the decay of the current (Fig. 6A&6B). The time course of the decay of the current trace was fitted with a single exponential function. The decay time constant (τ) for *I*_K_ was 145.9±11.9 ms in control and 63.4±4.9 ms in the presence of 100 µM (±) SKF83959 (n = 7, *P* = 0.0002, t = 6.42, df = 12, vs. control) (Fig. 6C). Moreover, as depicted in Fig. 6D, (±) SKF83959 suppressed the amplitude of tail current, but did not cause a crossover of the tail current.

(±) SKF83959 (100 µM) elicited a marked hyperpolarizing shift (nearly 20 mV) of the voltage dependence of steady-state activation curve of *I*_K_ (Fig. 7A). The value of *V*_1/2_ for activation was changed from −1.7±2.6 mV to −22.2±3.1 mV (n = 8, *P* = 0.0002, t = 5.10, df = 14), whereas the value of *k* for activation was from 16.8±0.9 to 13.6±1.1, n = 8, *P* = 0.038, t = 2.29, df = 14). (±) SKF83959 (100 µM) had no significant effect on its steady-state inactivation (Fig. 7B). The value of *V*_1/2_ for inactivation was nearly identical (−85.8±1.3 mV in control *vs*.−86.3±3.9 mV in the presence of (±) SKF83959, n = 8, *P* = 0.91, t = 0.11, df = 12). The value of *k* for inactivation has no significant change (−11.9±0.7 in control *vs*.−16.7±4.8 in the presence of (±) SKF83959, n = 8, *P* = 0.35, t = 1.00, df = 6). The same treatment accelerated the recovery of *I*_K_ from inactivation (Fig. 7C). The time constant of recovery was found to reduce from 294.1±30.4 ms to 107.7±10.4 ms (n = 6, *P* = 0.0002, t = 5.81, df = 10).

## Discussion

(±) SKF83959 is a putative phosphatidylinositol (PI)-linked D_1_-like receptor agonist [3], [4]. The agent has been shown to possess potent anti-parkinsonian effects in a variety of animal models for Parkinson's disease with less severe dyskinesia and motor fluctuation [6]–[8], [15], [18]. In the present study, we demonstrated for the first time that (±) SKF83959 and other substituted phenylbenzazepines inhibited the delayed rectifier K^+^ current (*I*_K_) in rat hippocampal neurons with the highest inhibition produced by (±) SKF83959. (±) SKF83959 is nearly 25-fold more potent than tetraethylammonium (TEA), a classical blocker of *I*_K_, which had an *IC*_50_ value of 1.05±0.21 mM in the same preparations [28].

To elucidate the mechanisms underlying (±) SKF83959-induced inhibition on *I*_K_, we found that the antagonists of D_1_, D_2_ or 5-HT_1A_ receptors did not block the inhibition of *I*_K_ by (±) SKF83959 (Fig. 3A), indicating that the inhibitory effect was a receptor-independent event. Furthermore, the fast nature in the onset of inhibition and recovery (Fig. 2B) implicates that the inhibition results from a direct interaction of (±) SKF83959 with the K^+^ channel. Moreover, we found that intracellular application of (±) SKF83959 had no effect on *I*_K_ (Fig. 4B), suggesting that the agent is most likely to act at the outer mouth of the pore of K^+^ channel. In an effort to determine how (±) SKF83959 blocks the K^+^ channel. We demonstrated that the inhibition of *I*_K_ by (±) SKF83959 was voltage-dependent (Fig. 5). Furthermore, the agent markedly accelerated the decay of *I*_K_ in addition to suppressing its amplitude (Fig. 6A), although lack of the crossover of tail current (Fig. 6D). The results suggest that the agent preferentially binds to the open state of the K^+^ channel [30], [31]. It is conceivable that (±) SKF83959 acts as an open-channel blocker at the delayed rectifier K^+^ channel. (±) SKF83959 probably binds to the K^+^ channel with 2∶1 stoichometry (Hill coefficient = 1.81±0.13).

Accumulating evidence shows that loss of intracellular K^+^ ions through enhanced delayed rectifier K^+^ channel (mainly Kv2.1 channel) mediates apoptosis of cortical neurons induced by a variety of treatments, such as serum deprivation, exposure to staurosporine or β–amyloid peptide fragment, etc. [23], [25]. In 6-hydroxydopamine -induced neurotoxicity, voltage-dependent potassium channels were found to play a vital role [32], since blockage of *I*_K_ by TEA was shown to effectively against the neurotoxicity and improve the neuronal viability. In other hand, the neuroprotective effects of classical *I*_K_ blocker TEA were also demonstrated in animal models of transient focal ischemia [26], [27]. Thus, the drug discovery targeted to specific K^+^ channel has been proposed as a potential therapeutic approach in treatment of neurodegenerative diseases [33]–[35]. We recently demonstrated that (±) SKF83959 protected rat cortical neurons against H_2_O_2_-induced injury, which was partially mediated though a putative PI-linked D_1_-like receptor-dependent mechanism via inhibition of GSK3β pathway [19]. The finding of (±) SKF83959 and its analogs as a potent blocker for the delayed rectifier K^+^ channel in the present study not only reveals a role of those compounds in the modulation of K^+^ channel, but also suggests a potential new mechanism for the neuroprotection of phenylbenzazepine derivatives. In the case of (±) SKF83959, how the neuroprotective effects via blockage of the delayed rectifier K^+^ channel contributes to the observed attenuation in the development of dyskinesia and reduction in the occurrence of motor fluctuation and wearing-off dyskinesia in chronic (±) SKF83959-treated PD animals [7], [18] are worth of further study.
